# Supplementary material for: Performance of Asphalt Rubber Mixture Overlays to Mitigate Reflective Cracking
Source: Materials (Basel). 2022 Mar 23;15(7):2375. doi: 10.3390/ma15072375 (PMC8999683; doi:10.3390/ma15072375)
Supplement: Supplementary file 1 [file materials-15-02375-s001.zip › materials-1632691-supplementary.pdf]

materials

MDPI

Supplement Materials

Figures and procedure

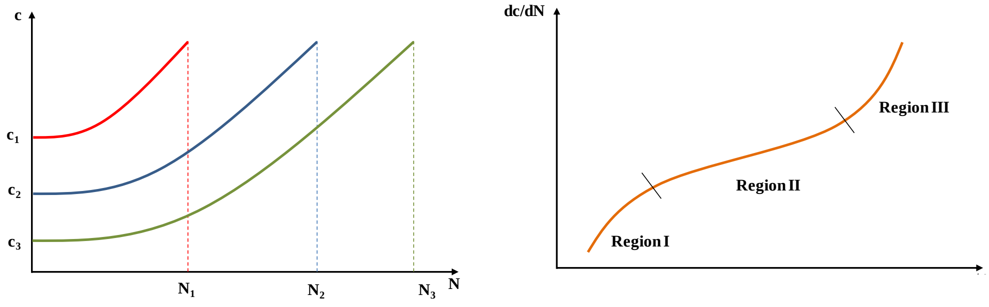

Figure S1. Crack length *versus* N and regions definition. **(a)** Crack length *versus* N. **(b)** Crack growth regions. (Based on [8]).

Asphalt rubber production

The asphalt rubber production was carried out by crumb rubber and asphalt mi × ing for 90 min at 180°C. The mechanical stirrer that promoted the incorporation and interactions between asphalt and rubber consists of a motor to which a propeller is coupled and a heating vessel monitored by a temperature controller (Figure S2). The rotation speed of the propeller varied from 250 to 350 rotations per minute. It was chosen to provide a homogeneous mi × ture between the crumb rubber and the asphalt without the segregation or sedimentation of the crumb rubber particles. The following procedure is illustrated in Figure S3:

1.First, weighing of the rubber to be added (percentage concerning the weight of asphalt base);

2. Preheating (digestion temperature minus 10°C) of the asphalt in the container, previously weighed, in an oven;

3. Heating the asphalt in the heating at to digestion temperature;

4. With the asphalt at the digestion temperature into the container (Figure S2a), the crumb rubber is adding (Figure S2b);

5. Crumb rubber addition into the asphalt at the mi × ing temperature under constant agitation (Figure S2c);

6. The asphalt rubber was ready after 90 min (Figure S2d).

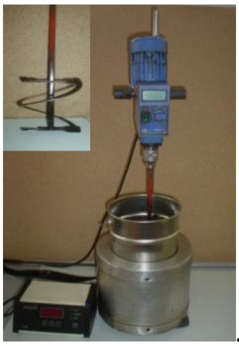.

Figure S2. Apparatus to produce asphalt rubber at the laboratory.

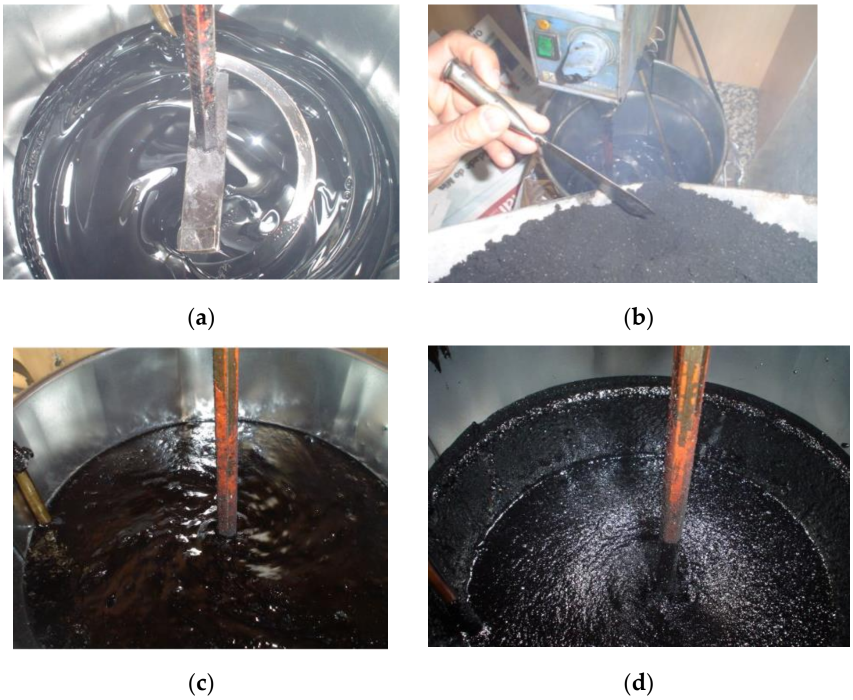

Figure S3. Asphalt rubber production. (**a**) Asphalt into the container. (**b**) Crumb rubber addition. (**c**) Asphalt mi × ing with crumb rubber. (**d**) Asphalt rubber.

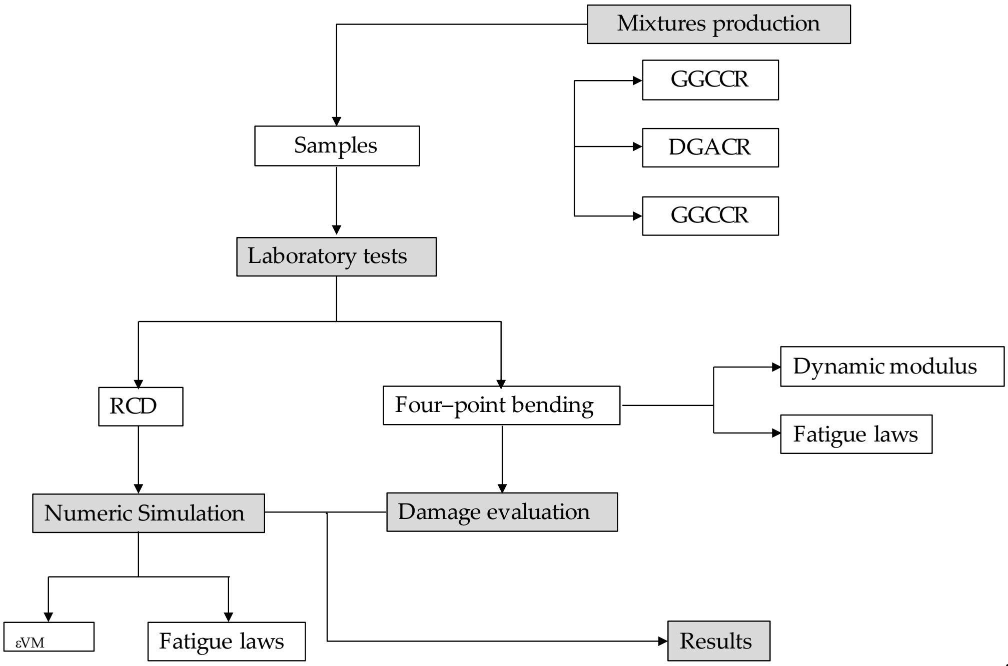

Figure S4. Flowchart of the e × perimental program.

Asphalt mi × tures production

Figure S5 illustrates the process of mi × tures producing, performed in the mechanical mi × er. After production, the mi × tures were compacted in a metal mould (75 × 49 × 8 cm^3) with a smooth roller (type WACKER RS 800 with a total mass of 861 kg), Figure S6a. Compaction was accomplished until the apparent design density was reached. The compaction followed AASHTO PP3/94 standard and the slabs remained in the mould (Figure S6b) at least 24 hours before demolding. From the slabs, samples were obtained to perform

the fatigue and modulus tests. The slabs were sawn (Figure S7a) to obtain nine prismatic samples, for each mi × ture, with the average dimensions: 381 ± 6.35 mm in length, 50.8 ± 6.35 mm in height and 63.5 ± 6.35 wide (Figure S7b).

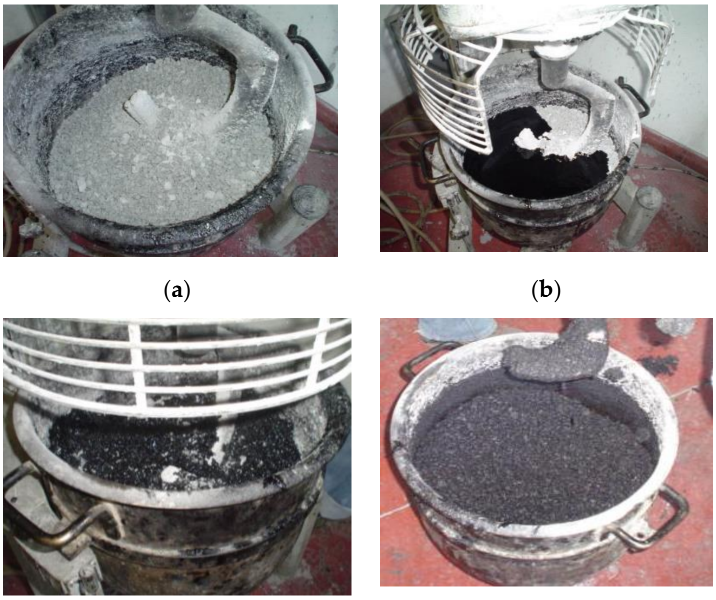

(**a**) (**b**)

(**c**) (**d**)

Figure S5. Roller and slab. (**a**) Aggregates introduced. (**b**) Asphalt addition. (**c**) Mi × ing. (**d**) Asphalt mi × ture into the slab.

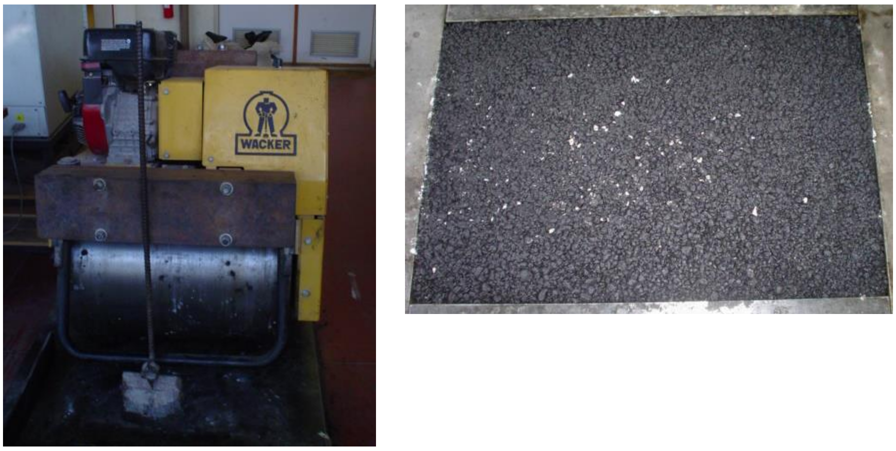

(**a**) (**b**)

Figure S6. Roller and slab. (**a**) Smooth roller. (**b**) Asphalt mixture into the slab.

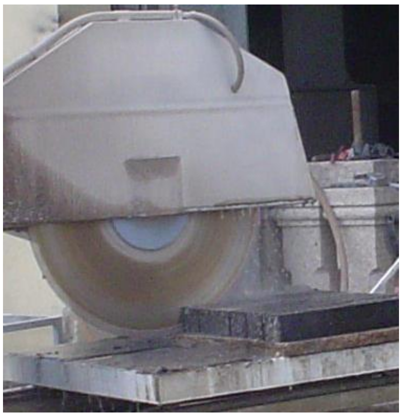

(a)

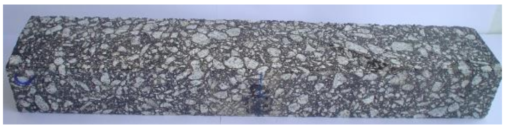

(b)

Figure S7. Samples obtaining. (**a**) Slab during the sawn. (**b**) Prismatic sample.

Numerical simulation

As for the numerical study, the adopted mesh for refle × ive cracking analysis represents an e × isting pavement. A crack was simulated through an element and a layer on top of the e × isting pavement representing an overlay. This mesh was described in other works developed by our group. The finite element model used in the numerical thermal analysis was performed using a general finite element analysis source code, ANSYS 7.0. It was a 3–D transient analysis using a standard finite element discretization in space. In the thermal finite–element mesh design, mesh compatibility with other mechanical models was observed. The designed mesh has 13,538 elements. A 3–D solid element, SOLID70, was used for three–dimensional thermal analysis. According to the previous e × planation, this element can have three–dimensional thermal conductions when applicable to a three–dimensional transient thermal analysis. The element has eight nodes with a single degree of freedom, defined as temperature, at each node. The thermal properties of pavement material, such as thermal conductivity, specific heat and density for each pavement layer, were defined in this element's "material properties" when the developed model. The model considers the e × istence of total friction as the interface between old and new pavement layers. The old layer was modelled with ten cracks, numbered from 1 to 10, starting from the model's left side (Figure S8). The distance between cracks was set to 10 cm, and the crack width was 3 mm. The model can quickly eliminate cracks to create any cracking configuration with any cracking spacing, from the pavement with only one crack to up to 10 cracks.

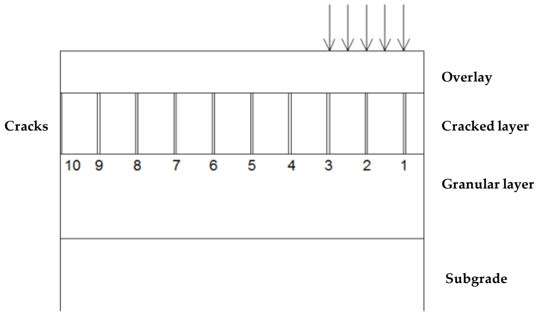

Figure S8. Schematic representation of the finite elements model (Based on [18]).

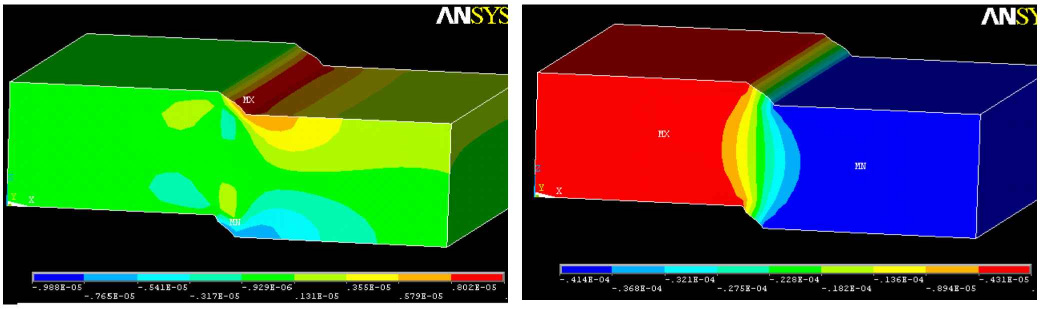

(**a**) (**b**)

Figure S9. Horizontal and vertical displacements. (**a**) Horizontal. (**b**) Vertical

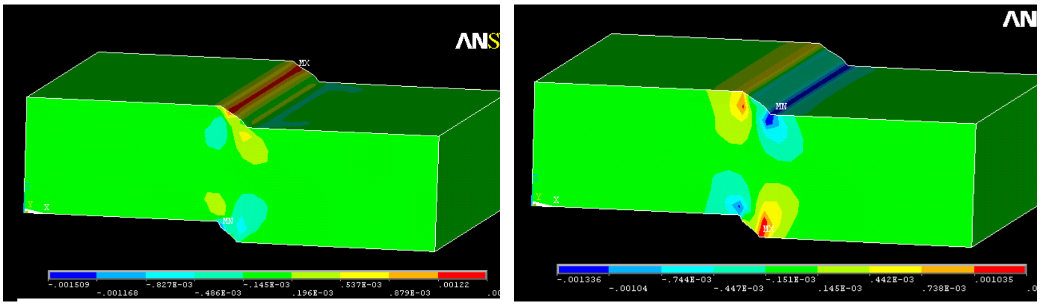

(**a**) (**b**)

Figure S10. Horizontal and vertical strains. (**a**) Horizontal. (**b**) Vertical

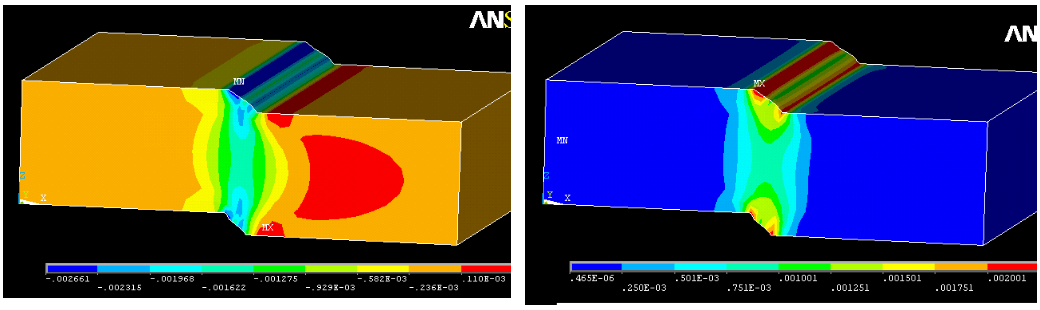

(**a**) (**b**)

Figure S11. Distortion and Von Mises strain. (**a**) Distortion. (**b**) Von Mises strainl.

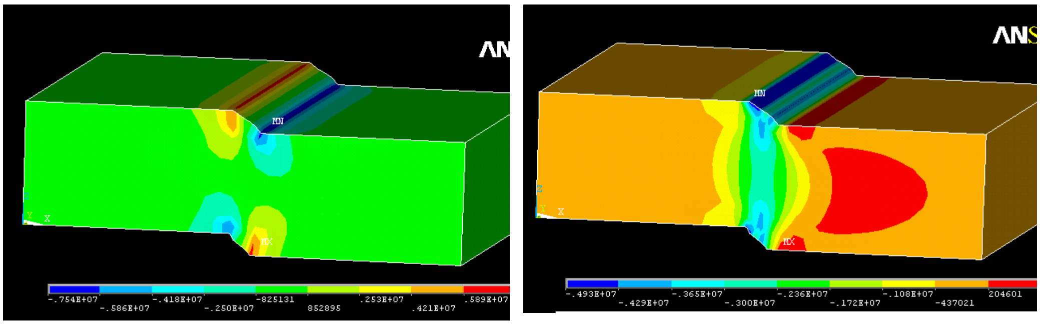

(**a**) (**b**)

Figure S12. Vertical and horizontal tension. (**a**) Horizontal. (**b**) Vertical.

Tables

Table S1. Annual traffic (Based on [18]).

A × le Load (kN)	$ESAL_{80kN}$
20 – 30	1,997,851
30 – 40	130,468
40 – 50	99,957
50 – 60	94,146
60 – 70	92,291
70 – 80	84,085
80 – 90	37,504
90 – 100	40,353
100 – 110	16,233
110 – 120	28,851
120 – 130	14,745
130 – 140	16,736
140 – 150	3189
150 – 160	3561
160 – 170	3411

Table S2. Overlay monthly damage.

Month	$T_{ma\times}$ [1]	ΔT [2]	Mi × Tures		
			GGGCR	DGACR	CONV
**July**	**10.1**	8.1	1.16×10^{-4}	6.07×10^{-5}	7.49×10^{-4}
August	11.0	10.7	1.05×10^{-4}	4.61×10^{-5}	7.43×10^{-4}
September	11.7	9.9	1.55×10^{-4}	1.06×10^{-4}	1.03×10^{-3}
October	15.7	13.1	3.20×10^{-4}	3.15×10^{-4}	2.19×10^{-3}
November	19.7	12.3	9.99×10^{-4}	6.58×10^{-4}	6.01×10^{-3}
December	28.1	15.2	2.83×10^{-3}	5.27×10^{-4}	1.70×10^{-2}
January	27.7	15.2	2.67×10^{-3}	6.24×10^{-4}	1.60×10^{-2}
February	25.8	11.5	3.75×10^{-3}	1.11×10^{-3}	1.95×10^{-2}
March	25.2	16.4	1.40×10^{-3}	3.17×10^{-4}	9.27×10^{-3}
April	16.75	9.1	8.65×10^{-4}	3.47×10^{-4}	4.79×10^{-3}
May	10.1	9.1	9.42×10^{-5}	4.64×10^{-5}	6.39×10^{-4}
June	9.1	9.4	5.61×10^{-5}	2.84×10^{-5}	4.03×10^{-4}

Table S3. RCD test results.

Mi × Ture	Sample	Vertical Displacement (mm)	Horizontal Force (N)	E × Perimental Coefficients					Life
				a	b	c	d	e	
**CONV**	**1**	**0.03**	**300**	-3.26×10^{4}	1.22×10^{5}	-8.63×10^{4}	3.67×10^{4}	-9.26×10^{2}	3.89×10^{4}
	2	0.02	200	-5.49×10^{5}	1.56×10^{6}	-8.00×10^{5}	1.24×10^{5}	1.45×10^{3}	3.36×10^{5}
	3	0.04	400	-5.49×10^{3}	6.96×10^{3}	2.53×10^{4}	6.55×10^{3}	5.08×10^{1}	3.34×10^{4}
	4	0.02	200	-3.00×10^{5}	9.58×10^{5}	-4.66×10^{5}	7.94×10^{4}	7.69×10^{2}	2.72×10^{5}
	5	0.03	300	-9.89×10^{3}	3.48×10^{3}	6.94×10^{4}	3.13×10^{2}	8.35×10^{2}	6.41×10^{4}
	6	0.04	400	-3.60×10^{3}	1.02×10^{4}	-6.36×10^{2}	8.54×10^{3}	-1.82×10^{1}	1.45×10^{4}
GGCCR	1	0.05	400	-7.99×10^{4}	-2.18×10^{5}	7.28×10^{5}	1.86×10^{4}	1.29×10^{3}	4.50×10^{5}
	2	0.05	400	2.35×10^{4}	-1.40×10^{5}	1.85×10^{5}	3.68×10^{4}	-1.61×10^{2}	1.05×10^{5}
	3	0.05	400	7.30×10^{4}	-4.29×10^{5}	6.48×10^{5}	2.40×10^{4}	3.31×10^{2}	3.16×10^{5}
	4	0.03	400	1.70×10^{6}	-2.71×10^{6}	2.56×10^{6}	1.60×10^{4}	-9.83×10^{2}	1.57×10^{6}

	5	0.03	400	2.69×10^5	-1.99×10^6	3.15×10^6	1.04×10^5	-6.08×10^3	1.53×10^6
DGACR	1	0.05	400	-8.06×10^3	-6.42×10^4	1.93×10^5	1.41×10^4	6.20×10^2	1.35×10^5
	2	0.05	400	-1.83×10^4	-3.58×10^4	1.35×10^5	1.67×10^4	-1.32×10^2	9.75×10^4
	3	0.05	400	1.82×10^4	-1.81×10^5	3.17×10^5	-1.21×10^4	4.47×10^2	1.43×10^5
	4	0.04	400	-2.09×10^4	-6.32×10^4	1.84×10^5	1.34×10^4	1.97×10^2	1.13×10^5
	5	0.04	400	9.02×10^4	-7.05×10^5	9.85×10^5	1.40×10^4	7.69×10^2	3.85×10^5
	6	0.03	400	-5.57×10^5	7.70×10^5	1.24×10^5	3.34×10^4	1.61×10^2	3.71×10^5

Table S4. Von Mises strains (ε_{vm}).

**Mi × Tures**	**Samples**	**Vertical Displacement (mm)**	**Horizontal Force (N)**	**Modulus (MPa)**	**ε_{vm} (10^{-6})**
CONV	1	0.03	300	6314	1684
	2	0.02	200	6314	1122
	3	0.04	400	6314	2245
	4	0.02	200	6314	1122
	5	0.03	300	6314	1684
	6	0.04	400	6314	2245
GGCCR	1	0.05	400	5192	2805
	2	0.05	400	5192	2805
	3	0.05	400	5192	2805
	4	0.03	400	5192	1694
	5	0.03	400	5192	1694
DGACR	1	0.05	400	6273	2801
	2	0.05	400	6273	2801
	3	0.05	400	6273	2801
	4	0.04	400	6273	2245
	5	0.04	400	6273	2245
	6	0.03	400	6273	1699
